# Comparison of Chicken Cecal Microbiota after Metaphylactic Treatment or Following Administration of Feed Additives in a Broiler Farm with Enterococcal Spondylitis History

**DOI:** 10.3390/pathogens10081068

**Published:** 2021-08-23

**Authors:** Julia Hankel, Björn Bodmann, Matthias Todte, Eric Galvez, Till Strowig, Dimitri Radko, Ali Antakli, Christian Visscher

**Affiliations:** 1 Institute for Animal Nutrition, University of Veterinary Medicine Hannover, Foundation, Hannover, Bischofsholer Damm 15, D-30173 Hannover, Germany; christian.visscher@tiho-hannover.de; 2 Tierarztpraxis MMT, Leopoldstraße 116, D-06366 Köthen, Germany; bjoern.bodmann@tap-mmt.de (B.B.); matthias.todte@tap-mmt.de (M.T.); 3 Helmholtz Center for Infection Research, Inhoffenstraße 7, D-38124 Braunschweig, Germany; Eric.Galvez@helmholtz-hzi.de (E.G.); till.strowig@helmholtz-hzi.de (T.S.); 4 Hannover Medical School, Carl-Neuberg-Straße 1, D-30625 Hannover, Germany; 5 Elanco Animal Health GmbH, Werner-Reimers-Straße 2-3, D-61352 Bad Homburg, Germany; radko_dmytro@elanco.com (D.R.); ali_antakli@elanco.com (A.A.)

**Keywords:** 16S rRNA gene, beta diversity, bacterial pathogens, poultry

## Abstract

Minimizing the clinical signs of *Enterococcus cecorum* infections causing enterococcal spondylitis in broiler herds is successful when initiated as metaphylaxis in the first week of life. Mechanistically, either the *Enterococcus* species present at that time are reduced by antibiotic treatment or antibiotic treatment might induce changes in intestinal microbiota composition with an indirect and subsequent influence. The aim of the present study was to examine the cecal microbiota of chickens after administering lincospectin or different additives to evaluate whether these additives have lincospectin-like effects on microbiota. Therefore, 157,400 broiler chickens were reared in four chicken houses (~40,000 birds each) on a broiler farm with history of enterococcal spondylitis. Each flock was treated either with lincospectin or water soluble esterified butyrins, *Bacillus* (*B*.) *licheniformis* or palm oil was added via drinking water during the first days of life. Ten birds per house were dissected at days 11, 20 and 33 of life and cecal microbiota were analyzed (16S rRNA gene sequencing). Lincospectin treatment elicited significant changes in the cecal microbiota composition until slaughter age. Among the tested additives, effects of *B. licheniformis* on cecal microbiota composition were most similar to those seen after the treatment with lincospectin at day 11.

## 1. Introduction

*Enterococcus* (*E.*) *cecorum* infections with a clinical course have been increasingly reported in different countries worldwide [1,2,3], forming an important emerging disease in modern broiler chickens associated with arthritis and osteomyelitis and leading to high mortality rates [1,2,4]. Lesions of osteomyelitis of the caudal thoracic vertebrae compressing the spinal cord and/or arthritis of the hock, stifle and coxofemoral joints provoked clinical signs, and *E. cecorum* was always isolated from these lesions [2,3]. The disease has been called enterococcal spondylitis, based on the frequent isolation of *E. cecorum* from the lesions and necrosis and inflammation observed in the free thoracic vertebrae of affected birds [5]. *E. cecorum* with pathogenic genotypes were identified in the intestines of naturally infected birds as early as week one, in contrast to commensal *E. cecorum* strains that did not appear until week three [6]. This ability to colonize the gut early in life may provide pathogenic *E. cecorum* strains with a competitive advantage and potentiate dissemination throughout a flock [6].

The onset of clinical signs like paresis and lameness [2,3] usually occurs at the beginning of week three of life [4,6], resulting in up to 7% increased mortality [2,3]. Disease outbreaks caused by enterococci are considered opportunistic [7] but the exact origin, predisposing factors and pathogenesis of enterococcal-associated vertebral osteoarthritis are still largely unknown [7,8,9]. It is suggested that intestinal colonization, bacteremia and osteochondrosis dissecans of the free thoracic vertebra in early life are crucial for the pathogenesis of enterococcal spondylitis [6]. Common features in epidemiology and clinical presentation have led several authors to suggest mechanisms by which this gastrointestinal inhabitant enters the bloodstream. Disturbance of the normal gut microbial balance could increase numbers of *E. cecorum* in the chicken intestine, and thus its prevalence in the poultry house environment and invasion of the systemic circulation might occur subsequent to damage to the respiratory, intestinal or integumentary barriers [8]. It is suggested that entry of *E. cecorum* into the bloodstream may occur because of gastrointestinal stress [5] or following disruption of the gut mucosal barrier caused by a previous enteric disease, such as coccidiosis or bacterial enteritis [6,8], as well as mechanical or toxic irritants [6]. Recently, it could be shown that *Enterococcus* spp. might enhance the survival of other pathogenic intestinal bacteria and co-infections, leading to increased virulence [10]. In contrast, infections with other intestinal pathogens decrease bacteremia and spinal lesions caused by pathogenic *E. cecorum* [11]. These observations indicate interactions of *Enterococcus* spp. with other intestinal bacteria. Furthermore, even if it is suggested that disruption of the intestinal structure could potentiate *E. cecorum* bacteremia, it was not observed that clinical intestinal diseases necessarily cause *E. cecorum* bacteremia [6].

Diseased flocks are often treated with one or more antibiotics. However, the effect of antibiotic therapy is temporary [7] or only successful when initiated as metaphylaxis from the first week of life [2]. Additionally, several Enterococcus species seem to be resistant against the most frequently used antibiotics in poultry [12,13]. Against this background, mechanisms affecting *E. cecorum* other than the direct bactericidal or bacteriostatic ones could be suspected. This led to the assumption that early antibiotic treatment might induce substantial changes to the microbiota composition of the intestines with the consequence of an indirect and subsequent influence on this bacterial species and the course of the disease. As *E. cecorum*-positive spleens were found as early as week 1 and during weeks 2 and 3 respectively [6], the suspected most likely time window of bacterial translocation from the gut became the focus of the present investigation of intestinal microbiota composition (represented by day 11).

Antibiotics need to be used more prudently and alternatives are needed in animal agriculture, while high-throughput technologies could help to better understand effects and mechanisms of action of the various components guiding the selection of antibiotic alternatives [14]. Some used alternatives to antibiotics in agricultural animals are feed additives, which include prebiotics, probiotics, and organic acids [14]. Studies have shown that butyric acid preparations might be useful adjuncts to reduce necrotic enteritis in antibiotic-free broiler production [15]. Their mechanism of action against the disease must involve other host-mediated activities as there was no direct microbial inhibition of *Clostridium perfringens* [16]. Butyric acid is an important energy source for gut epithelial cells and stimulates epithelial cell proliferation and differentiation [17,18,19], it has well documented anti-inflammatory effects [20], promotes concentration-dependent intestinal barrier function [21,22] and demonstrates beneficial effects against intestinal bacteria with zoonotic relevance in poultry production [23,24]. Some commercially available probiotic *Bacillus* strains, including *Bacillus* (*B.*) *licheniformis*, were effective at inhibiting pathogenic *E. cecorum* in vitro [25] and the administration of *B. licheniformis* was able to normalize ileum microbiota disorders caused by necrotic enteritis in chickens [26]. Finally, plant oils containing medium-chain fatty acids showed in vitro antibacterial activity against gram-positive intestinal bacteria, such as *E. cecorum* while the same oils did not show any effect on commensal bacteria (*Bifidobacterium* spp. and *Lactobacillus* spp.) [27].

The aim of the present investigation was to examine microbiota changes due to the metaphylactic administration of lincospectin, and to ascertain whether using water soluble esterified butyrins, *B. licheniformis* or palm oil have lasting effects on the cecal microbiota of broiler chickens similar to those seen after treatment with lincospectin.

## 2. Results

### 2.1. General Health and Performance Parameters

The fattening period in chicken houses one, three and four ran without complications. At day 28 of life, chickens in chicken house two (esterified butyrins) were treated with colistin sulfate (Colistinsulfat 100, bela-pharm GmbH & Co. KG, Vechta, Germany, 300 g/1000 L) for three days because of a systemic *E. coli* infection.

Average body weights measured at the slaughterhouse (total body weight of chickens divided by their number) met breeder performance objectives with the exception of chicken house 3 and 4 at days 28 and 33, which remained below expectations (Table 1).

### 2.2. 16S rRNA Gene Analyses

After the filtering step, two further samples were removed from the dataset because of high dissimilarity to all other samples at day 33. Therefore, 115 of the obtained 120 samples were included in the statistical analyses of microbiota. The dataset contained 2,216,872 reads (mean number of reads: 19,277; range: 10,344–95,833) mapped to 541 operational taxonomic units (OTUs).

At day 11, the contribution of the factor treatment was highest, explaining 25.2% of the sample variability (Table 2). With increasing age, the influence of this factor on the microbial composition of the samples decreased.

**Figure 1:**
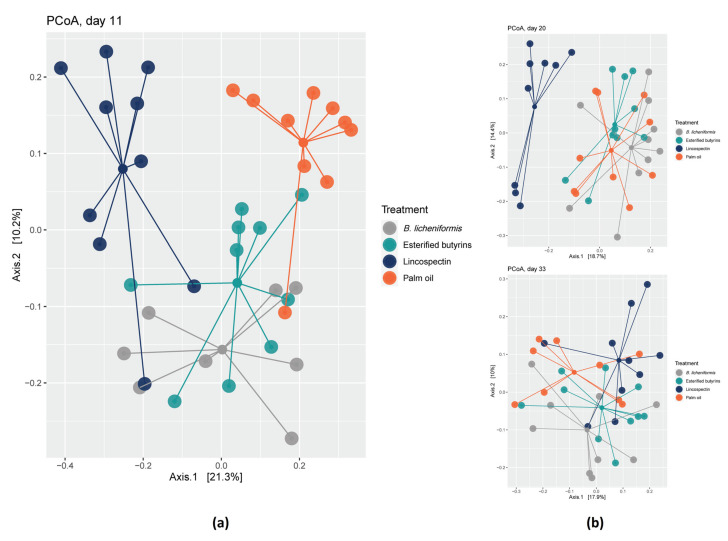
Bray–Curtis dissimilarity-based principal coordinate analysis (PCoA) was performed on cecal samples collected at: (**a**) day 11, (**b**) days 20 and 33. Each point represents a different bird; colored lines connect birds of one treatment (*B. licheniformis*, esterified butyrins, lincospectin and palm oil).

The microbiota composition of the cecal samples of the birds treated with lincospectin differed from the three additives. Bray–Curtis distances revealed that with increasing chicken age or time after treatment, dissimilarity in cecal microbiota composition between chickens treated with lincospectin and the other chickens decreased (Table 3, Figure 1). Chickens that had received *B. licheniformis* showed the highest similarity with lincospectin-treated chickens at day 11, followed by chickens treated with esterified butyrins.

**Figure 2:**
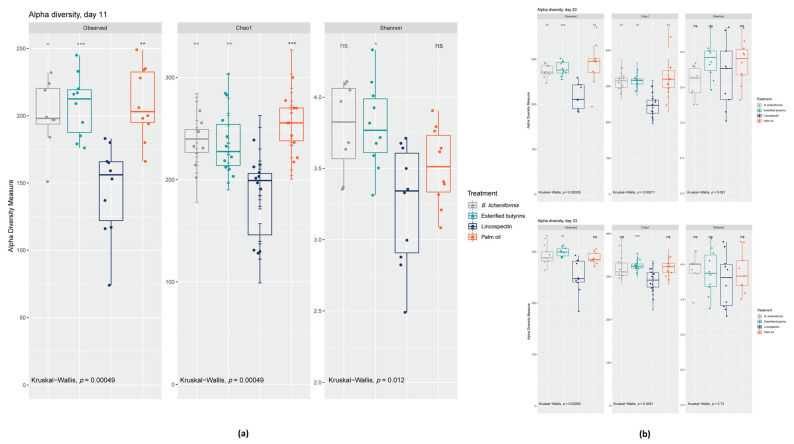
Boxplots showing alpha diversity in cecal samples using the species richness estimators observed species, Chao1 and Shannon index at: (**a**) day 11, (**b**) days 20 and 33 (ns: *p* > 0.05, *: *p* ≤ 0.05, **: *p* ≤ 0.01, ***: *p* ≤ 0.001).

Comparison of the measured species richness estimators, observed species, Chao 1 and Shannon index in the cecal contents of the birds is shown in Figure 2. The treatment with lincospectin during the first days of the chicken’s life lowered species richness significantly compared to the administered additives until day 33, while significant effects of lincospectin on species evenness was only seen at day 11. At day 11, the Shannon index demonstrated that richness and evenness of cecal bacteria in chickens that had received *B. licheniformis* and esterified butyrins were highest. Nevertheless, species richness estimators revealed no statistically significant differences between the three additives at any of the measured time points (Table S1).

**Figure 3:**
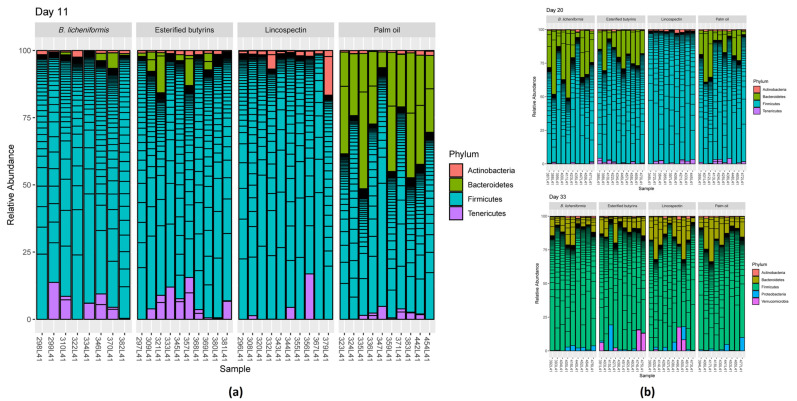
Bar charts represent the relative abundances of the dominant phyla comprising 50 of the most dominant OTUs in cecal samples of chickens at an age of (**a**) 11, (**b**) 20 and 33 days.

At day 11, the microbiota of the cecal contents was dominated at the phylum level by *Firmicutes*, *Tenericutes* and *Bacteroidetes*. Relative abundance of *Bacteroidetes* was the highest and relative abundance of *Firmicutes* was the lowest in the cecal samples of the chickens that had received palm oil (Figure 3), resulting in the lowest *Firmicutes* to *Bacteroidetes* ratio. The cecal microbiota of chickens in the other groups were dominated by *Firmicutes*. The statistical analyses for the differences in bacterial family abundance revealed that chickens treated with lincospectin showed reduced abundance of the families *Bacteroidaceae* (OTU_2), uncultured rumen bacterium (OTU_57, Order: *Mollicutes* RF9), *Defluviitaleaceae* (OTU_277), Ambiguous_taxa (OTU_121, Order: *Clostridiales*), and *Christensenellaceae* (OTU_102) in comparison to the groups receiving one of the three feed additives (Table S2 and Figure S1). The greatest influence of lincospectin was seen on the family *Bacteroidaceae* showing an up to 4096-fold lower abundance compared to administering palm oil. After administering the feed additives several families belonging to the order *Clostridiales* (class: *Clostridia*, phylum: *Firmicutes*) were enriched compared to lincospectin treatment. In contrast, chickens that were treated with lincospectin showed an enriched abundance of the families *Bifidobacteriaceae* (OTU_76) and *Staphylococcaceae* (OTU_208) compared to all three administered feed additives. *Bifidobacteriaceae* abundance became 62-fold (palm oil), 276-fold (*B. licheniformis*), and 413-fold higher (esterified butyrins) due to the lincospectin treatment.

At day 20, relative abundance of *Bacteroidetes* in the cecal contents of the chickens that had received *B. licheniformis* and esterified butyrins increased, while the cecal microbiota of the lincospectin-treated chickens was still dominated by the phylum *Firmicutes* (Figure 3). At day 33, relative abundance of *Firmicutes* in cecal samples of chickens treated with lincospectin had decreased while *Bacteroidetes* increased. This development is reflected in a shift of the *Firmicutes* to *Bacteroidetes* ratio giving a similar picture between the groups at day 33 (Figure 3).

Members of the bacterial family *Enterococcaceae* were present from day 11 at the latest. With regard to the relative abundance of the family *Enterococcaceae,* no differences between the groups were found at day 11 (Table 4).

## 3. Discussion

Due to the observed effect of the metaphylactic use of lincospectin by poultry veterinarians to prevent enterococcal spondylitis (personal communication), the present study aimed to first describe the intestinal microbiota composition under this treatment before comparing it with the intestinal microbiota of chickens after administering different feed additives.

### 3.1. Microbiota Composition under Lincospectin Treatment

The administration of all treatments occurred only during the first days of the chicken’s life, but the microbiota composition was influenced significantly until slaughter age. At day 11, the factor treatment explained 25.2% of the variability in microbial composition and persisted until slaughter age (about 30 days after withdrawal of the treatments), where the factor treatment still explained 16.7% of the microbial composition variability. PERMANOVA revealed that antibiotic treatment (low-dose concentration) of turkey poults caused a change in community composition at day 6, to a lesser extent than in the present study (R^2^ = 0.151, *p* = 0.001) [29]. Antibiotic treatment with lincospectin and colistin enteromix of six-month old pigs induced significant differences in community structure as well (beta diversity of OTUs was assessed by PERMANOVA), whereby only 1.8% of sample’s variability could be explained by this factor (R^2^ = 0.018, *p* = 0.037) [30]. Age at the time of antibiotic administration appears to play a role in the extent to which administration affects intestinal microbiota composition. Single early-life antibiotic administration can alter the microbiota that persist long after exposure has ceased [31].

Richness in cecal samples of lincospectin-treated chickens was significantly affected, showing a lower richness compared to the other additives until slaughter age. This species-reducing effect of antibiotic treatment is a widely reported [32,33,34] and therefore expected effect. Evenness of the present bacterial species was less affected with advanced age as indicated by the Shannon index. This indicates that the cecal contents of lincospectin-treated chickens hosted less varied bacterial species but with similar even distribution compared to the other chickens that had received one of the three additives. The cecal microbiota of chickens treated with lincospectin had not only an overall lower richness but also a clear dominance of *Firmicutes* with low proportions of *Bacteroidetes* until at least day 20. Similar results have already been shown in humans where the level of functional diversity is linked to the relative abundance of *Bacteroidetes*, and microbiota enriched for *Firmicutes*/*Actinobacteria* have a lower level of functional diversity [35]. Diversity of the cecal microbiota in the present study increased generally with increasing age. This development was expected as the early cecal microbiota is characterized by poor diversity but gains complexity and matures to a stable and diverse microbiota with increasing age [36]. The number of genera found in the chicken ceca doubled from day 7 to day 42 [37]. The cecal microbiota of chickens are more susceptible to changes as they are developing during half of the production period [36]. For this reason, an accelerated development of chicken gut microbiota can be considered beneficial with regard to resistance to several intestinal pathogens or the outcome of infectious diseases. This could be shown already for *Salmonella* Enteritidis [38] and *Campylobacter jejuni* [23,39]. The treatment with lincospectin in the present study seemed to retard this microbiota development. With regard to potentially pathogenic *Enterococcus* (*E.*) *cecorum* strains, this effect of lincospectin on microbiota development should be less advantageous, which does not correspond to the effects seen in practice (personal communication with poultry veterinarians). Diseased flocks are often treated with one or more antibiotics. However, the effects of administering antibiotics seem to be only successful when initiated as a metaphylaxis from the first week of life onwards [2]. This supports the suspected importance of the early period of the life of broiler chickens in the pathogenesis of enterococcal spondylitis [6]. Therefore, it can be assumed that pathogenic *E. cecorum* strains appear to be distinct from the other chicken intestinal pathogens, which generally colonize broiler flocks later (in the case of *Campylobacter jejuni* with an age of 3–4 weeks [40,41]) or only interact with a certain bacterial community.

### 3.2. Microbiota Maturation under Administering of Palm Oil

The microbiota composition of chickens that had received palm oil was the one with the largest difference in the microbiota composition of chickens that had been treated with lincospectin. At day 11, the relative abundance of *Firmicutes* and *Bacteroidetes* in chickens that had received palm oil clearly differed from all other groups. The *Bacteroidetes* to *Firmicutes* ratio changes with age, as seen in the present study. *Proteobacteria* is the first most abundant phylum in the cecal contents of chickens (the remaining part of cecal microbiota is formed by representatives of the family *Lachnospiraceae*), while within the following days, the families *Lachnospiraceae* and *Ruminococcaceae* (phylum *Firmicutes*) gain predominance, which is in turn taken over by *Bacteroidetes* with advanced age, reaching a constant ratio of *Bacteroidetes* and *Firmicutes* formed by equal numbers of the representatives of both phyla in adult chickens [42,43,44]. Even if *Firmicutes* and *Bacteroidetes* are usually equally represented in the cecal microbiota of healthy adult chickens, there is still high individual variation and individual chickens with 10% to 90% *Bacteroidetes* in their microbiota exist without exhibiting any signs of abnormal behavior [42]. The major families from *Firmicutes* colonized chicken ceca are *Lachnospiraceae* and *Ruminococcaceae*, followed by *Lactobacillaceae*, *Veillonellaceae* and *Erysipelotrichaceae* [42]. The abundance of the families *Lachnospiraceae* and *Erysipelotrichaceae* were enriched in the lincospectin-treated chicks compared to chicks that had received palm oil (*p* < 0.05, Table S2). All members of the family *Lachnospiraceae* are anaerobic, fermentative, and chemoorganotrophic, while some have strong hydrolyzing activities, e.g., through carbohydrate-active enzymes [45]. Therefore, *Lachnospiraceae* have a considerable capacity to utilize diet-derived polysaccharides (including starch, inulin, and arabinoxylan) and further degrade components of plant material (cellulose and hemicellulose) which are fermented and converted to short-chain fatty acids (SCFAs) like acetate, butyrate, and propionate [46,47]. The SCFAs can be absorbed and used for energy by the host [47]. Additionally, SCFAs and the biosynthesis of bacterial metabolites from aromatic amino acid metabolism by *Lachnospiraceae* strengthens the intestinal barrier and are associated with the maintenance of gut health [46,47]. Within the phylum *Firmicutes*, members of the families *Lachnospiraceae* and *Ruminococcaceae* have been shown to express enzymes favoring the production of butyrate over propionate, while representatives of the phylum *Bacteroidetes* are mainly propionate producers [43]. Several genus belonging to the family *Erysipelotrichaceae* are capable of fermentation of carbohydrates to butyrate [42]. Butyrate has direct trophic effects, improves epithelial integrity and defense systems and has also been implicated in the down-regulation of bacterial virulence, which is why butyrate is a helpful feed additive in animal production, especially when ingested soon after birth, as it controls gut health disorders caused by bacterial pathogens (reviewed in Guilloteau et al. [48]).

The aerobic atmosphere of hatcheries, farms and animal houses can contain spores of *Clostridiales*, families *Lachnospiraceae* and *Ruminococcaceae*, which is why both families colonize soon after *E. coli* in newly hatched chicks [42]. The likelihood of a chicken being colonized by *Bifidobacteriaceae* is low and therefore a longer time is needed for their appearance in gut microbiota [42]. About three days post hatch *Bifidobacteriaceae* rise is thought to be an important step in the maturation of cecal microbiota as it stimulates the growth of *Bacteroidaceae* [36]. Even if the treatment with lincospectin seemed to retard microbiota development in the present study, the abundance of *Bifidobacteriaceae* was significantly enriched in lincospectin-treated chickens compared to chickens that had received one of the other feed additives at day 11. *Bifidobacteriaceae* play an important role in pathogen exclusion and intestinal barrier function, with most of the evidence coming from mammalian studies [36], while in chickens the role of *Bifidobacterium* has not yet been fully elucidated and the benefit of cecum *Bifidobacteriaceae* colonization in chickens needs to be further clarified [49,50]. *Bifidobacterium* species are not numerically dominant in chickens [42,49], but are used as probiotics mainly in humans but also in chickens [51]. After in ovo supplementation of Bifidobacteria strains, serum amino transferases were not affected which led the authors to the assumption that this observation might be connected with the reduction effect of Bifidobacteria on the translocation of harmful bacteria in the gut and liver [52]. Since the invasion of pathogenic *E. cecorum* strains into the systemic circulation is thought to occur subsequent to the disruption of the gut mucosal barrier [6,8], altering intestinal microbiota composition during the first days of life in favor of bacterial families which promote intestinal barrier integrity should be beneficial and represent a possible mechanism of action of lincospectin against this disease.

It can be supposed that adding palm oil to drinking water during the first days of the chicken’s life promoted microbiota maturation compared to the other additives and the treatment with lincospectin, which might be a disadvantage with regard to potentially pathogen *E. cecorum* strains. Nevertheless, it should be emphasized that the possible effects of the additives under *E. cecorum* infection were not evaluated in the present study, no statement can be made in this respect.

### 3.3. Similar Microbiota Composition in Chickens Administered B. Licheniformis, Esterified Butyrins and Lincospectin

Among the tested additives, the effects of *B. licheniformis* on cecal microbiota composition were most similar to those seen after the treatment with lincospectin at day 11. Administering the probiotic strain *B. licheniformis* did not result in differed cecal bacterial diversity compared to administering esterified butyrins or palm oil but induced a clear dominance of *Firmicutes* with low proportions of *Bacteroidetes* at day 11, this being very similar to the esterified butyrins and lincospectin-treated groups. Similarly, this observation of the abundance of the phylum *Firmicutes* and of the genus *Lactobacillus* in excreta increased in broilers fed *B. licheniformis*–fermented products, whereas the abundance of the phyla *Verrucomicrobia* and *Bacteroidetes* decreased in response to the treatment [53]. Trela et al. [54] were equally unsurprised about the diversity indices being lower after *B. licheniformis* supplementation because of the induced increase in the proliferation of *Firmicutes*. Administering *B. licheniformis* seems to induce a modulation of the pH value within the gastrointestinal tracts of chickens [54,55], which is suspected to be a cause of the induced alterations in microbiota composition by *B. licheniformis* [54]. In the present study, members of the bacterial family *Enterococcaceae* were present from day 11 at the latest. No differences in the relative abundance of *Enterococcaceae* were found between the groups at day 11, whereby relative abundance in chickens that had received *B. licheniformis* were the lowest. Additionally, Chen, Ying-Chu and Yu [53] observed a decreased abundance of the genus *Enterococcus* in the excreta of broilers fed *B. licheniformis*-fermented products. Additionally, some commercially available probiotic *Bacillus* strains were effective at inhibiting pathogenic *E. cecorum* in vitro [25]. Considering these observations with the results of the present study, this feed additive is very interesting for studying *E. cecorum* infection with a standardized approach.

## 4. Materials and Methods

### 4.1. Experimental Design, Animals and Housing, Sampling

In total, 157,400 broiler chickens of the breed Ross 308 were supplied as day-old chicks from the same hatchery and reared on one fattening farm in Germany with enterococcal spondylitis history in four chicken houses (~40,000 birds each) for about five weeks (37 to 38 days). One identical conventional complete diet was offered *ad libitum* in all chicken houses. Chickens in each chicken house were treated either with lincospectin or one of three different additives administered via drinking water during the first days of life. Chickens in the first chicken house were treated with lincospectin (Lincospectin^®^ Pulver, Zoetis Deutschland GmbH, Berlin, Germany, 250 g/1000 L) at the first day of life up to the age of two days (three days in total). At the first day of life up to the age of three days (four days in total), water soluble esterified butyrins were administered to the chickens in the second chicken house (0.12–0.24 mL/kg body weight). Over the same period, the chickens in the third chicken house were offered drinking water containing *Bacillus licheniformis* (70 g/1000 L), while palm oil (2 L/1000 L) was given to the chickens in the fourth chicken house during the first seven days of life.

Ten birds per chicken house were dissected at days 11, 20 and 33 of life. The animals were killed without interventions before being dissected. Anesthesia and the killing of the birds were carried out in accordance with Annex 2 (to Section 2, paragraph 2) of the Regulations on the Welfare of Animals Used for Experiments or for Other Scientific Purposes (TierSchVersV). Anesthesia was performed by head stroke. After bleeding, cecal contents were removed under sterile conditions, placed in reaction vessels, immediately frozen and stored at −80 °C until simultaneous analysis.

### 4.2. 16S rRNA Gene and Statistical Analyses

Microbiota analyses were performed as already described by Hankel et al. [23]. Samples were purified (Kit: BS 365, BioBasic Inc., Markham, ON, Canada) before the hypervariable region V4 of the 16S rRNA gene was amplified in accordance with previously described protocols [56] using primer F515/R806. Amplicons were sequenced on the Illumina MiSeq platform (PE250). The Usearch8.1 software package (http://www.drive5.com/usearch/ accessed on 22 May 2018) was used to assemble, quality control and cluster obtained reads; -fastq_mergepairs –with fastq_maxdiffs 30 was used to merge the reads. Chimeric sequences were identified and removed with the help of cluster_otus (-otu_radius_pct 3) and the Uchime command included in the Usearch8.1 workflow. Quality filtering was set up with the fastq_filter (-fastq_maxee 1); minimum read length, 200 bp before reads were clustered into 97% ID operational taxonomic units (OTUs). The UPARSE algorithm [57] was used to determine the OTU clusters and representative sequences. Silva database v128 [58] and the RDP Classifier [59] was used for taxonomic assignment with a bootstrap confidence cutoff of 70%. Data visualization and statistical analyses of microbiota were performed with R (version 4.0.3, www.r-project.org accessed on 15 January 2021) and the R-package “phyloseq” (version 1.32.0, https://joey711.github.io/phyloseq/ accessed on 15 January 2021) [60]. OTUs that were not present in at least one sample were pruned. Additionally, OTUs with an abundance < 0.02% were filtered and finally, samples with fewer than 999 total reads were removed. Reads assigned to *Cyanobacteria*, *Chloroplast* and *Mitochondria* were filtered.

Samples were normalized (transformed to relative abundance) prior to ordination. Factors contributing to the differences in microbial composition of the samples were identified with Permutational multivariate analysis of variance (PERMANOVA) on Bray–Curtis distances. Ordination was performed using the Bray–Curtis dissimilarity-based principal coordinate analysis (PCoA). Pairwise comparisons for Bray–Curtis distances of bacterial communities associated with treatments were tested using Analysis of similarities (ANOSIM) within the R-package “vegan” (version 2.5.6, https://rdrr.io/cran/vegan/ accessed on 8 August 2021) [61]. The species richness estimators Observed Species, Chao 1 and Shannon index were used to measure sample diversity. A Kruskal–Wallis rank sum test was performed to compare the sample diversity indices of all groups, while additionally each of the groups were compared pairwise to the reference group (lincospectin-treated chickens) using the Wilcoxon rank sum test with the *p*-value adjustment method: holm. Relative abundances of the 50 most abundant OTUs belonging to bacterial phyla were shown as bar charts for days 11, 20, and 33. R-package DESeq2 (version 1.33.4, http://bioconductor.org/packages/release/bioc/vignettes/DESeq2/inst/doc/DESeq2.html accessed on 8 August 2021) [62] was used to find taxa with significantly different abundance using the Wald test for significance testing, while *p*-values were adjusted by the Benjamini and Hochberg (BH) method to control the false discovery rate (FDR) of 5%. Relative abundances of the dominant families within the phyla *Bacteroidetes* and *Firmicutes*, which at the same time also showed significant differences between the lincospectin-treated and non-treated chickens, and families that were significantly enriched in lincospectin-treated chickens compared to all three other groups were shown.

## 5. Conclusions

It can be concluded that the microbiota composition in the present study was influenced lastingly until slaughter age by the treatment of lincospectin only within the first days of life. At day 11, the microbiota composition of chickens treated with *B. licheniformis* were most similar to chickens treated with lincospectin, immediately followed by chickens treated with esterified butyrins, making these two additives among the tested ones the most interesting for further studies. Nevertheless, it should be underlined that the potential of *B. licheniformis* and water soluble esterified butyrins to prevent enterococcal spondylitis has still to be proven. Future studies with regard to pathogen enterococcal strains might focus on the parameters of enterococcal colonization, intestinal bacteria and intestinal barrier in the early life stages of chickens. A better understanding of these interactions could help in the development of strategies to prevent or treat this disease.

## Figures and Tables

**Table 1:** Average body weights of chickens (g) measured at slaughterhouse after depletion of chicken houses in batches and at the end of the fattening period.

Day of Life	Chicken House 1	Chicken House 2	Chicken House 3	Chicken House 4
28	1538 (*n* = 7285)	1559 (*n* = 7113)	1457 (*n* = 7025)	
33	1977 (*n* = 4958)	1984 (*n* = 4954)	1920 (*n* = 5006)	1902 (*n* = 8255)
37	2350 (*n* = 25,025)	2424 (*n* = 23,854)		
38			2444 (*n* = 24,677)	2467 (*n* = 28,224)

Breeder performance objectives: 1501 g at day 28, 1956 g at day 33, 2334 g at day 37 and 2429 g at day 38 [28].

**Table 2:** PERMANOVA testing the factor treatment on microbial composition of cecal samples taken at days 11, 20 and 33.

	Day 11		Day 20		Day 33	
	R^2^	Pr(>F)	R^2^	Pr(>F)	R^2^	Pr(>F)
Treatment	0.2520	0.001	0.2430	0.001	0.1667	0.001

**Table 3:** ANOSIM results (Statistic R values with a range from 0 (similar) to 1 (dissimilar)) on Bray–Curtis distances between bacterial communities associated with treatments based on pairwise test.

		*B. licheniformis*	Esterified Butyrins	Palm Oil
**Lincospectin**	Day 11	0.4110 ***	0.4778 ***	0.7518 ***
	Day 20	0.7355 ***	0.6055 ***	0.5668 ***
	Day 33	0.1854 ***	0.1770 ***	0.3005 ***

*** Significance value: *p* ≤ 0.001.

**Table 4:** Relative abundances (mean %, ± sd) of selected bacterial families in cecal samples taken at day 11.

Day 11				
Family	*B. licheniformis*	Esterified Butyrins	Lincospectin	Palm Oil
*Bacteroidaceae*	0.955 ± 1.41	3.15 ± 3.96	0.010 ± 0.007	24.7 ± 11.1
*Ruminococcaceae*	57.0 ± 13.5	57.5 ± 6.10	41.1 ± 10.7	45.9 ± 9.60
*Lachnospiraceae*	17.8 ± 14.7	14.2 ± 4.77	29.5 ± 10.8	12.5 ± 4.22
*Erysipelotrichaceae*	3.38 ± 1.89	4.64 ± 3.60	13.2 ± 13.0	3.29 ± 1.77
*Bifidobacteriaceae*	0.005 ± 0.011	0.005 ± 0.009	1.94 ± 3.54	0.026 ± 0.063
*Staphylococcaceae*	0	0.002 ± 0.004	0.179 ± 0.209	0.011 ± 0.017
*Enterococcaceae*	0.031 ± 0.035	0.158 ± 0.157	0.207 ± 0.172	0.110 ± 0.231

## Data Availability

The data presented in this study are openly available in NCBI BioProject repository (https://www.ncbi.nlm.nih.gov/bioproject/PRJNA741093 accessed on 24 June 2021), reference number (PRJNA741093).

## Supplementary Materials

The following are available online at https://www.mdpi.com/article/10.3390/pathogens10081068/s1, Table S1. *p*-values ^1^ of pairwise comparisons of species richness estimators in cecal samples of chickens, Table S2. Families with significant different abundance between lincospectin and the administered feed additives at day 11, Figure S1. Volcano plot showing families with significant different abundance (FDR-adjusted *p*-values < 0.01) and absolute log2 fold change >2 between chickens treated with Lincospectin and: (**a**) *B. licheniformis*, (**b**) esterified butyrins or (**c**) palm oil, at day 11.
