# Supplementary material for: Comparison of Chicken Cecal Microbiota after Metaphylactic Treatment or Following Administration of Feed Additives in a Broiler Farm with Enterococcal Spondylitis History
Source: Pathogens. 2021 Aug 23;10(8):1068. doi: 10.3390/pathogens10081068 (PMC8398815; doi:10.3390/pathogens10081068)
Supplement: Supplementary file 1 [file pathogens-10-01068-s001.zip › Supplementary material.pdf]

Table S1. *p*-values [1] of pairwise comparisons of species richness estimators in cecal samples of chickens.

**Day 11**			
**Observed Species index**	***B. licheniformis***	**Esterified butyrins**	**Lincospectin**
**Esterified butyrins**	1		
**Lincospectin**	**0.0116**	**0.0002**	
**Palm oil**	1	1	**0.0029**
**Chao 1 index**	***B. licheniformis***	**Esterified butyrins**	**Lincospectin**
**Esterified butyrins**	0.9654		
**Lincospectin**	**0.0082**	**0.0075**	
**Palm oil**	0.4294	0.4294	**0.0005**
**Shannon index**	***B. licheniformis***	**Esterified butyrins**	**Lincospectin**
**Esterified butyrins**	0.9650		
**Lincospectin**	0.0780	**0.0310**	
**Palm oil**	0.3030	0.2520	0.3810
**Day 20**			
**Observed Species index**	***B. licheniformis***	**Esterified butyrins**	**Lincospectin**
**Esterified butyrins**	0.7680		
**Lincospectin**	**0.0019**	**0.0003**	
**Palm oil**	0.3910	0.7680	**0.0039**
**Chao1 index**	***B. licheniformis***	**Esterified butyrins**	**Lincospectin**
**Esterified butyrins**	1		
**Lincospectin**	**0.0016**	**0.0022**	
**Palm oil**	1	1	**0.0026**
**Shannon index**	***B. licheniformis***	**Esterified butyrins**	**Lincospectin**
**Esterified butyrins**	0.0540		
**Lincospectin**	1	1	
**Palm oil**	0.1160	1	1
**Day 33**			
**Observed Species index**	***B. licheniformis***	**Esterified butyrins**	**Lincospectin**
**Esterified butyrins**	0.4085		
**Lincospectin**	**0.0395**	**0.0026**	
**Palm oil**	0.5354	0.0710	**0.0710**
**Chao1 index**	***B. licheniformis***	**Esterified butyrins**	**Lincospectin**
**Esterified butyrins**	0.5470		
**Lincospectin**	0.1739	**0.00045**	
**Palm oil**	1	1	0.0507
**Shannon index**	***B. licheniformis***	**Esterified butyrins**	**Lincospectin**
**Esterified butyrins**	1		
**Lincospectin**	1	1	
**Palm oil**	1	1	1

[1] *p*-values < 0.05 are written in bold

Table S2. Families with significant different abundance between lincospectin and the administered feed additives at day 11.

**Lincospectin vs *B. licheniformis***							
**log2FoldChange**	**pvalue**	**padj**	**OTU_ID**	**Phylum**	**Class**	**Order**	**Family**
−6.87	7.15e-15	2.29e-13	OTU_2	Bacteroidetes	Bacteroidia	Bacteroidales	Bacteroidaceae
8.11	7.94e-10	1.27e-08	OTU_76	Actinobacteria	Actinobacteria	Bifidobacteriales	Bifidobacteriaceae
6.85	2.29e-09	2.44e-08	OTU_208	Firmicutes	Bacilli	Bacillales	Staphylococcaceae
−5.34	1.76e-08	1.41e-07	OTU_137	Firmicutes	Clostridia	Clostridiales	Clostridiaceae 1
−10.1	3.82e-08	2.44e-07	OTU_57	Tenericutes	Mollicutes	Mollicutes RF9	uncultured rumen bacterium
−7.38	3.32e-07	1.77e-06	OTU_277	Firmicutes	Clostridia	Clostridiales	Defluviitaleaceae
−5.50	1.16e-05	5.32e-05	OTU_121	Firmicutes	Clostridia	Clostridiales	Ambiguous_taxa
−5.96	8.96e-05	0.0004	OTU_148	Tenericutes	Mollicutes	Mollicutes RF9	uncultured bacterium
2.43	0.0011	0.0040	OTU_24	Proteobacteria	Gammaproteobacteria	Enterobacteriales	Enterobacteriaceae
−4.90	0.0016	0.0052	OTU_74	Tenericutes	Mollicutes	Mollicutes RF9	Ambiguous_taxa
−0.951	0.0020	0.0059	OUT_3	Firmicutes	Clostridia	Clostridiales	Ruminococcaceae
−2.69	0.0032	0.0084	OTU_102	Firmicutes	Clostridia	Clostridiales	Christensenellaceae
−2.11	0.0040	0.0097	OTU_115	Firmicutes	Clostridia	Clostridiales	Clostridiales vadinBB60 group
−2.02	0.0066	0.0220	OTU_62	Firmicutes	Bacilli	Lactobacillales	Streptococcaceae
2.44	0.0147	0.0461	OTU_11	Firmicutes	Erysipelotrichia	Erysipelotrichales	Erysipelotrichaceae
**Lincospectin vs Esterified butyrins**							
**log2FoldChange**	**pvalue**	**padj**	**OTU_ID**	**Phylum**	**Class**	**Order**	**Family**
−8.57	1.46e-18	6.27e-17	OTU_2	Bacteroidetes	Bacteroidia	Bacteroidales	Bacteroidaceae
−11.5	2.99e-15	6.43e-14	OTU_57	Tenericutes	Mollicutes	Mollicutes RF9	uncultured rumen bacterium
8.69	1.44e-13	2.07e-12	OTU_76	Actinobacteria	Actinobacteria	Bifidobacteriales	Bifidobacteriaceae
6.52	7.58e-10	8.15e-09	OTU_208	Firmicutes	Bacilli	Bacillales	Staphylococcaceae
−4.51	1.15e-07	9.93e-07	OTU_227	Firmicutes	Clostridia	Clostridiales	Clostridiaceae 1
−6.15	2.79e-06	2.00e-05	OTU_277	Firmicutes	Clostridia	Clostridiales	Defluviitaleaceae
−5.52	5.95e-06	3.66e-05	OTU_67	Tenericutes	Mollicutes	Mollicutes RF9	uncultured bacterium
−5.22	7.10e-06	3.82e-05	OTU_74	Tenericutes	Mollicutes	Mollicutes RF9	Ambiguous_taxa
−5.59	2.04e-05	9.76e-05	OTU_121	Firmicutes	Clostridia	Clostridiales	Ambiguous_taxa
−2.53	0.0021	0.0090	OTU_102	Firmicutes	Clostridia	Clostridiales	Christensenellaceae
0.986	0.0025	0.0099	OTU_4	Firmicutes	Clostridia	Clostridiales	Lachnospiraceae
**Lincospectin vs Palm oil**							
**log2FoldChange**	**pvalue**	**padj**	**OTU_ID**	**Phylum**	**Class**	**Order**	**Family**

−12.0	4.91e-100	1.47e-98	OTU_2	Bacteroidetes	Bacteroidia	Bacteroidales	Bacteroidaceae
−7.64	9.98e-19	1.50 e-17	OTU_121	Firmicutes	Clostridia	Clostridiales	Ambiguous_taxa
−7.37	1.25e-09	1.25e-08	OTU_67	Tenericutes	Mollicutes	Mollicutes RF9	uncultured bacterium
−9.23	1.03e-07	7.74e-07	OTU_57	Tenericutes	Mollicutes	Mollicutes RF9	uncultured rumen bacterium
5.95	2.24e-06	1.19e-05	OTU_76	Actinobacteria	Actinobacteria	Bifidobacteriales	Bifidobacteriaceae
−4.94	2.39e-06	1.19e-05	OTU_227	Firmicutes	Clostridia	Clostridiales	Clostridiaceae 1
−5.73	2.17e-05	9.28e-05	OTU_277	Firmicutes	Clostridia	Clostridiales	Defluviitaleaceae
−4.75	3.65e-05	0.0001	OTU_205	Tenericutes	Mollicutes	Mollicutes RF9	Ambiguous_taxa
3.63	0.0001	0.0005	OTU_208	Firmicutes	Bacilli	Bacillales	Staphylococcaceae
−3.03	0.0008	0.0025	OTU_102	Firmicutes	Clostridia	Clostridiales	Christensenellaceae
−2.40	0.0036	0.0098	OTU_744	Bacteroidetes	Bacteroidia	Bacteroidales	Prevotellaceae
−0.766	0.0053	0.0145	OTU_1	Firmicutes	Clostridia	Clostridiales	Ruminococcaceae
1.48	0.0106	0.0268	OTU_11	Firmicutes	Erysipelotrichia	Erysipelotrichales	Erysipelotrichaceae
0.726	0.0212	0.0499	OTU_4	Firmicutes	Clostridia	Clostridiales	Lachnospiraceae
