# Supplementary figures and images for: Comparison of Chicken Cecal Microbiota after Metaphylactic Treatment or Following Administration of Feed Additives in a Broiler Farm with Enterococcal Spondylitis History
Source: Pathogens. 2021 Aug 23;10(8):1068. doi: 10.3390/pathogens10081068 (PMC8398815; doi:10.3390/pathogens10081068)

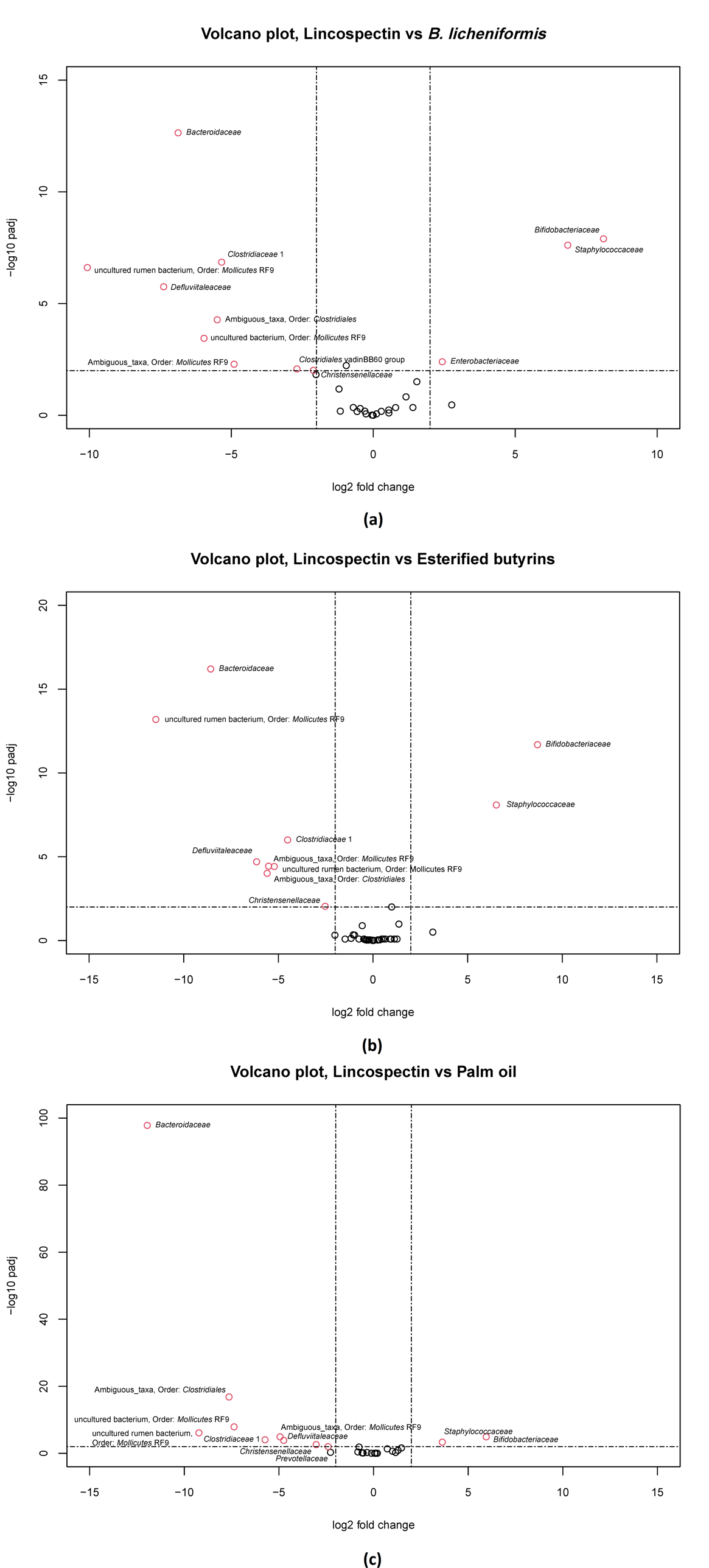

Supplement: Supplementary file 1 [file pathogens-10-01068-s001.zip › pathogens-1292367-Figure S1. Volcano plot showing families with significant different abundance (FDR-adjusted p-values 0.01) and absolute log2 fold change 2 between chickens treate.tif]
